# Collagen VI in healthy and diseased nervous system

**DOI:** 10.1242/dmm.032946

**Published:** 2018-05-31

**Authors:** Ilaria Gregorio, Paola Braghetta, Paolo Bonaldo, Matilde Cescon

**Affiliations:** Department of Molecular Medicine, University of Padova, 35131 Padova, Italy

**Keywords:** Central nervous system, Collagen VI, Peripheral nervous system

## Abstract

Collagen VI is a major extracellular matrix protein exerting a number of functions in different tissues, spanning from biomechanical to regulatory signals in the cell survival processes, and playing key roles in maintaining the stemness or determining the differentiation of several types of cells. In the last couple of years, emerging findings on collagen VI have led to increased interest in its role in the nervous system. The role of this protein in the peripheral nervous system was intensely studied and characterized in detail. Collagen VI acts as a regulator of Schwann cell differentiation and is required for preserving peripheral nerve myelination, function and structure, as well as for orchestrating nerve regeneration after injury. Although the role and distribution of collagen VI in the peripheral nervous system is now well established, the role of this distinctive extracellular matrix component in the central nervous system, along with its links to human neurological and neurodegenerative disorders, remains an open field of investigation. In this Review, we summarize and discuss a number of recent findings related to collagen VI in the central and peripheral nervous systems. We further link these findings to different aspects of the protein that are relevant to human diseases in these compartments in order to provide a comprehensive overview of the roles of this key matrix component in the nervous system.

## Abstract (precis)

**Summary:** Although collagen VI was so far known to be mostly involved in skeletal muscle homeostasis, this Review summarizes a number of recent studies that highlight its key role in the peripheral and central nervous systems, pointing at a link with human neurological disorders.

## Introduction

**Fig. 1.**
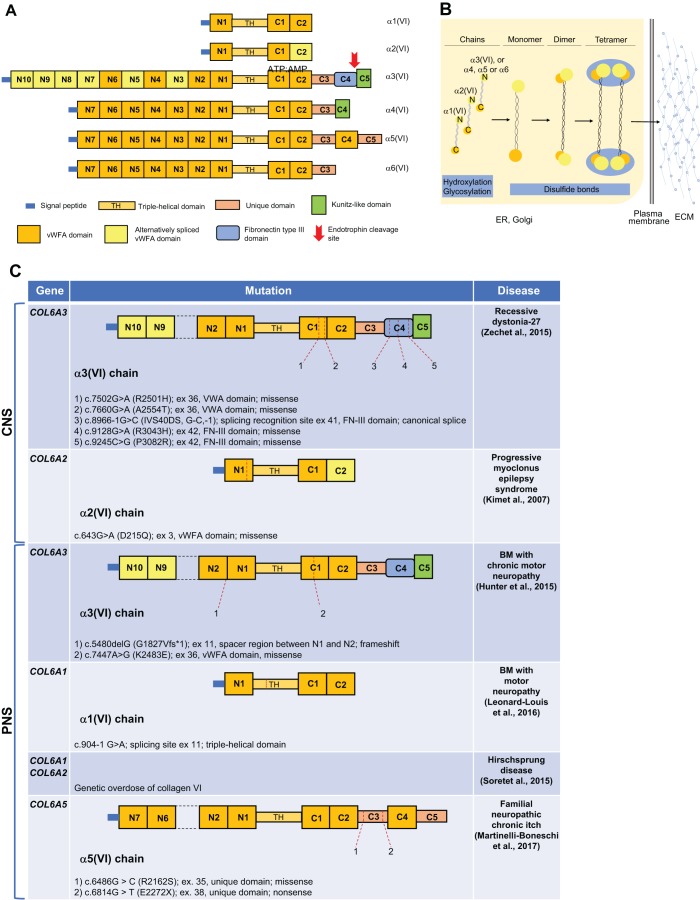
**ColVI structure, assembly and mutations linked to human nervous system diseases.** (A) Schematic representation of ColVI chains and their protein domains. (B) Diagram displaying ColVI assembly and secretion. (C) A summary of the mutations in *COL6* genes that were described to be linked to human disorders affecting the CNS and the PNS. BM, Bethlem myopathy; ER, endoplasmic reticulum; FN-III, fibronectin type III; TH, triple-helical domain; vWFA, von Willebrand factor type A.

Collagen VI (ColVI) is a broadly distributed extracellular matrix (ECM; see Glossary, Box 1) protein with a number of unique structural and functional features. It is considered a network-forming collagen, because it forms characteristic beaded microfilament nets (Fig. 1A) in the ECM (Keene et al., 1988), through which it is able to interact with several other ECM components and with cell membrane receptors. Being a member of the collagen family, ColVI finely modulates the stiffness and mechanical properties of the ECM. Interestingly, it also impacts several intracellular processes and pathways, such as apoptosis, autophagy, cell stemness (Box 1) and differentiation, tumor progression, and macrophage polarization (Box 1) (Cescon et al., 2015).

**Box 1. Glossary**

**Adventitia:** the outermost connective layer of a blood vessel. It is also called *tunica externa*.

**Amyloid angiopathy:** a disease characterized by deposition of amyloid deposits in the media and adventitia of cortical and leptomeningeal vessels. It can be associated with Aβ-42, Aβ-40, other Aβ isoforms or other proteins.

**Amyloid precursor protein:** the transmembrane protein whose cleavage generates Aβ polypeptide, involved in the pathogenesis of Alzheimer's disease.

**Apoptosis:** programmed cell death.

**Aqueous humor:** the liquid produced by the ciliary epithelium of the lens, filling the anterior and posterior chambers within the eye.

**Autophagy:** an intracellular bulk degradation process leading to the destruction of proteins or unnecessary or dysfunctional cellular components.

**Basal lamina:** a thin, planar specialized ECM, tethering cells to the underlying connective tissue.

**C-fibers:** small, unmyelinated sensory fibers characterized by a low conduction velocity. They react to thermal, mechanical or chemical stimuli.

**Choroid plexus:** a layer of cuboidal epithelial cells surrounding a core of capillaries and loose connective tissue found in the CNS ventricles. It produces the cerebrospinal fluid.

**Corpus callosum:** the myelinated commissure connecting the right and left cerebral hemispheres.

**Dentate gyrus:** the region of the hippocampus involved in the formation of memory and the site of neurogenesis.

**Diencephalon:** the portion of the forebrain localized rostrally to the midbrain, containing the thalamus and hypothalamus.

**Electromyography:** a technique that records the electrical activity of a muscle.

**Endoneurium, perineurium, epineurium:** sheaths of connective tissue enveloping single myelinating fibers (endoneurium), nerve fascicles (perineurium) and entire nerves (epineurium).

**Extracellular matrix:** a network of secreted proteoglycans and proteins, providing a physical scaffold as well as biochemical and biomechanical cues to cells in a tissue.

**Glia limitans:** astrocytic processes connected to the pia mater in the brain and spinal cord.

**Glia:** non-neuronal cells that support and regulate PNS and CNS functions.

***In situ* hybridization:** a probe hybridization-based technique that allows the localization of a specific mRNA in a tissue section.

**Insertional mutagenesis screening:** a screen performed by mutating a genome via random insertion of a DNA sequence, in order to identify genes linked to a particular phenotype.

***LacZ* reporter:** a genetic construct containing the bacterial *lacZ* gene (coding for the β-galactosidase enzyme) under the control of a gene promoter and/or regulatory region of choice. Upon addition of the substrate analog X-gal, a colored product is produced if the *lacZ* gene is active, allowing the detection of the promoter's or regulatory region's activity.

**Ledged-beam walking test:** a test used to detect and measure sensory-motor impairments in rodent models, by letting them walk on a suspended beam increasingly narrowing from the start to the end site.

**Luse bodies:** a form of aggregated ColVI fibrils with a high periodicity (40 to >100 nm), found in connective tissues under pathological conditions.

**Macrophage polarization:** the capacity of inactive macrophages to acquire different phenotypes depending on their microenvironment.

**Megacolon:** a massive, pathological enlargement of the colon.

**Meninges:** the membranous structures wrapping the brain and the spinal cord. Named pia mater, arachnoid and dura mater from the inner to the outer layer.

**Morpholino oligonucleotides:** oligomers composed of DNA bases attached to a phosphorodiamidate backbone, used to modify gene expression, e.g. by gene knockdown.

**Nerve conduction velocity:** the speed of an electric impulse along axons in a nerve.

**Neural crest:** the embryonic structure formed by neural crest cells that will give rise to diverse cell types such as melanocytes, smooth muscles, glia.

**Neural interstitial matrix:** the loose ECM present in the CNS parenchyma.

**Neural tube:** the embryonic precursor of the central nervous system in vertebrates.

**Nociception:** a neural process involving the transmission and processing of noxious stimuli.

**Nodes of Ranvier:** periodic myelin interruptions occurring along an axon. The distance between Ranvier nodes is called internodal length.

**Parenchyma:** the functional tissue characteristic of an organ. Brain parenchyma is composed of neurons and glial cells.

**Perineuronal nets:** specialized ECM found around certain neuronal bodies, regulating synaptic stability.

**Psammoma body:** a round laminar structure composed of calcium apatite and collagen, which can be found in meningiomas.

**Pseudogene:** a gene that has lost at least some functionality, relative to the complete gene, in its expression or protein coding ability, often resulting from the accumulation of multiple mutations.

**Schwann cells:** the major glial cells of the PNS. They wrap peripheral axons with myelin, a substance composed of lipids and proteins, thus ensuring a fast, saltatory electric impulse. The CNS counterparts of Schwann cells are oligodendrocytes.

**Sclerotome:** the embryonic structure that will give rise to vertebrae and intervertebral discs. Through the ventral sclerotome, neural crest cells migrate to form Schwann cells, dorsal root and sympathetic ganglia.

**Stemness:** the ability of a cell to self-renew and maintain a wide differentiation potential.

**Stoichiometric ratio:** a stable proportion of molecules interacting or reacting with each other.

**Wallerian degeneration:** a process occurring after a nerve injury, characterized by the distal degeneration of the axons, myelin clearance and macrophage or microglia infiltration.

For many years, ColVI has been described as being composed of three chains, α1(VI), α2(VI) (both ∼140 kDa) and α3(VI) (∼250 kDa), encoded by the *COL6A1*, *COL6A2* and *COL6A3* genes, respectively (Chu et al., 1987; Colombatti et al., 1987). However, further studies examining genomic databases identified three additional ColVI chains, named α4(VI) (∼250 kDa), α5(VI) (∼290 kDa) and α6(VI) (∼250 kDa), encoded by the *COL6A4*, *COL6A5* and *COL6A6* genes (Fitzgerald et al., 2008). These share homology with the α3(VI) chain (Gara et al., 2008). In humans, the *COL6A4* gene is inactivated, as it underwent an inversion during primate evolution, generating two separate nonprocessed pseudogenes (Box 1) (Fitzgerald et al., 2008; Gara et al., 2008). The different ColVI chains share some common features, such as a relatively short triple-helical (or collagenous) domain and two large globular N- and C-terminal regions. These contain variable numbers of von Willebrand factor type A (vWFA) modules (Fig. 1A), making the structure of ColVI unique among the collagens (Chu et al., 1988, 1990a; Bonaldo et al., 1989, 1990; Bonaldo and Colombatti, 1989). ColVI chains are also characterized by the presence of conserved cysteine residues in the triple-helical domain and at the junctions between the globular regions and the triple helix, forming a number of disulphide bonds required for chain assembly into monomers, dimers and tetramers. Indeed, ColVI has a distinctive multistep process of intracellular assembly in which three chains assemble into triple-helical monomers of ∼500 kDa (Fig. 1B). These monomers assemble with a 1:1:1 stoichiometric ratio (Box 1) between the main three chains (α1, α2 and α3), where the α3(VI) chain can be substituted by one of the newly identified ones in specific tissues (Sabatelli et al., 2011). Disulphide bonds then allow the formation of antiparallel dimers (∼1000 kDa) and finally tetramers (∼2000 kDa) (Fig. 1B) (Colombatti et al., 1987, 1995; Colombatti and Bonaldo, 1987; Chu et al., 1990b). After post-translational modifications in the Golgi apparatus, the ColVI tetramers are secreted into the ECM, where they form the typical 100-nm periodic end-to-end beaded microfilaments (Fig. 1B) (Bruns et al., 1986; Engvall et al., 1986).

Owing to its causative role in several congenital muscular dystrophies, collectively named ColVI-related myopathies and including Bethlem myopathy (BM), Ullrich congenital muscular dystrophy (UCMD) and myosclerosis myopathy (Lampe and Bushby, 2005; Merlini et al., 2008; Bönnemann, 2011), ColVI has been thoroughly investigated in skeletal muscles. Therefore, the amount of clinical, functional and molecular data currently available for ColVI in skeletal muscle is much larger than that for other tissues (Cescon et al., 2015). Nonetheless, a number of recent findings shed new light on the role of ColVI in the nervous system and will be discussed in this Review. These recent findings were based on studies in animal models (Box 2) and genetic analyses in humans, linking ColVI gene variants to disorders of the nervous system. In this Review, we first provide an overview of previous and new findings on ColVI in the central nervous system (CNS), as well as its roles and functions in the peripheral nervous system (PNS). We then discuss the growing evidence of ColVI links with CNS diseases, highlight the several hints of its involvement in PNS disorders, and finally comment on its contribution to nervous system tumors.

**Box 2. Animal models revisited: an accent on the nervous system**

Although largely used to investigate the myopathic phenotype induced by ColVI deficiency, genetically engineered animal models harboring mutant ColVI were recently able to reveal novel roles of this ECM component in the nervous system.

In particular, ColVI null (*Col6a1*^−/−^) mice represent a peerless tool, not only as a valuable model for ColVI-related myopathies and for dissecting the critical functions of this ECM molecule in skeletal muscles, but also for investigating the roles of ColVI in other tissues (Cescon et al., 2015). The *Col6a1*^−/−^ mouse model bears a targeted inactivation of the *Col6a1* gene, thus preventing ColVI assembly and deposition within the ECM (Bonaldo et al., 1998). The use of *Col6a1*^−/−^ mice unveiled novel aspects of ColVI biology in the CNS and PNS, as discussed in the main text (Cheng et al., 2009; Chen et al., 2014, 2015; Cescon et al., 2016). Other mouse models for ColVI-related myopathies were generated, such as those targeting the *Col6a3* gene. In particular, Pan and colleagues generated two transgenic mouse lines, one (*Col6a3^hm/hm^*) characterized by a hypomorphic *Col6a3* allele with a deletion of the sequences coding for a region important for stabilizing the triple helical monomer (Pan et al., 2013), and a second one (*Col6a3^+/d16^*) harboring a deletion of exon 16 (Pan et al., 2014). Two additional mouse lines were generated by targeting *Col6a2* (Solares Perez et al., 2012; Mohassel et al., 2015).

Zebrafish models for ColVI deficiency were also developed. Using morpholino oligonucleotides that target either exon 9 or exon 13 of the zebrafish *col6a1* ortholog to knock down mRNA expression, Dowling and colleagues showed that the morphant fish, those with reduced *col6a1* expression, display a myopathic phenotype (Telfer et al., 2010). The images provided in their paper showed defects that were more extensive than merely muscle malformations (Telfer et al., 2010). Although defects of the CNS and PNS were not considered in the study, the authors did describe significant motor deficiencies that cannot exclude neurological deficits. In order to overcome the limitations of transient knockdown with morpholinos, and to model and analyze later stages in disease, a zebrafish line with a mutation in the *col6a1* gene was recently generated by targeting the splice donor site of intron 14, resulting in an in-frame skipping of exon 14 of the corresponding mRNA (Radev et al., 2015). Beside the presence of abnormal myofibers and the ultrastructural defects characteristic of ColVI-related myopathies, this zebrafish line also displayed erratic behaviors, suggested to be caused by oxygen intake deficits and hypoxia (Radev et al., 2015). Such abnormal behaviors were elsewhere linked to anxiety (Egan et al., 2009), thus suggesting novel hypotheses for CNS involvement.

Other ColVI genes, besides *col6a1*, were also targeted in zebrafish. Telfer and colleagues previously demonstrated that morpholino-mediated knockdown of *col6a3* exon 9 recapitulated the myopathic phenotype of *col6a1* morphants (Telfer et al., 2010). Intriguingly, a recent study used morpholino-mediated disruption of *col6a3* exon 42 in zebrafish embryos to corroborate data collected from human patients affected by dystonia. They demonstrated that the morphants had axonal outgrowth deficits without a primary muscular involvement (Zech et al., 2015). Similar findings concerning altered axonal growth were demonstrated in *col6a2* and in *col6a4* zebrafish morphants, which also displayed motor activity abnormalities and muscle defects (Ramanoudjame et al., 2015).

The various findings collected from the animal models described above emphasize the importance of *in vivo* studies for unraveling the novel roles of ColVI in specific tissues, such as the nervous system, and for revealing unexpected phenotypical outcomes of targeted mutations. The development and use of tissue-specific *in vivo* models, such as conditional knockouts of the *Col6* genes in neurons and glial cells and CRISPR/Cas9-mediated genome editing, will certainly shed more light on the *in vivo* roles of ColVI in the nervous system.

## ColVI in the CNS

The ECM plays multiple roles in the different compartments of the CNS. Indeed, in the CNS, the ECM is found in the connective tissue, mainly consisting of the basal lamina associated with the meninges and the brain blood vessels, as well as in the nervous tissue itself, in the form of perineuronal nets and the neural interstitial matrix dispersed in the parenchyma (Box 1) (Lau et al., 2013). ColVI was reported to be present in both the CNS basal lamina and nervous tissue parenchyma. Immunohistochemical analyses initially associated ColVI deposition with brain blood vessels in human CNS samples. In particular, ColVI has been found in the adventitia (Box 1) of the meningeal vessels and of the larger intraparenchymal arteries and veins, but not in cortical vessels, including capillaries (Roggendorf et al., 1988). The same study also detected abundant deposition of this ECM protein in the choroid plexus (Box 1), in the superficial glia (Box 1) and in cranial nerves (Roggendorf et al., 1988). ColVI deposition in the CNS is dynamically regulated during development. At 21 weeks of gestation, ColVI-positive vessels were observed by immunohistochemistry in the basal ganglia and deep white matter, while from 38 weeks of gestation onwards, the presence of ColVI becomes restricted to the cerebral cortex and the superficial white matter (Kamei et al., 1992).

ColVI also plays a key role in the development of the meninges. A study highlighting the importance of the ECM in the proper organization of the glia limitans (Box 1) described the ability of meningeal cells to secrete ColVI (Sievers et al., 1994). Further studies in mice revealed the presence of *Col6a1* transcripts in the meninges in all the developmental stages included in the analysis, from embryonic day (E) 11.5 to postnatal life (Marvulli et al., 1996). In agreement with this, another study showed that the mRNA and protein products for the three main ColVI chains (Fig. 1A) are present in murine meninges during embryogenesis, mostly from E12.5 to E14.5 (Dziadek et al., 1996). These data indicate an important role for meningeal cells in secreting and depositing a highly specialized ECM in the nervous system, and point at a role for ColVI in organizing such a matrix. Interestingly, ColVI expression was also observed in the mesenchyme of the developing murine choroid plexus at E14 (Dziadek et al., 1996), underscoring a role of ColVI not only in CNS basal lamina organization, but also in modulating neural tissue homeostasis.

Concerning the presence of ColVI in the nervous tissue proper, its source and regulation are still an open field of investigation. Studies of transgenic mouse lines expressing the *lacZ* reporter (Box 1) fused with various fragments of the *Col6a1* regulatory regions upstream of the transcription start site (TSS), revealed that the transcriptional control of the *Col6a1* gene is remarkably complex and dynamic, with several regulatory elements arranged in a modular manner across a locus of more than 10 kb. These regulatory elements confer in a spatially and temporally restricted expression (Braghetta et al., 1996). The same study identified one regulatory element located between 4.0 and 5.4 kb upstream of the TSS that confers *Col6a1* expression in peripheral nerves, as well as one enhancer located between 6.2 and 7.5 kb upstream of the TSS regulating *Col6a1* expression in the meninges. Although a previous northern blotting-based study did not detect expression of ColVI in the murine CNS (Marvulli et al., 1996), *Col6a1* expression was subsequently identified in the CNS parenchyma of the transgenic *lacZ* mouse lines discussed above. At the time, a possible explanation for this discrepancy suggested that repressive elements, which would normally repress *Col6a1* expression in the brain and thus corroborate the findings in Marvulli et al. (1996), were absent from the sequences used for generating the different *lacZ* transgenic mouse lines (Braghetta et al., 1996).

A recent body of work, reporting ColVI expression in the brain and its implications in CNS diseases, prompted renewed interest in this protein. In particular, a key study demonstrated that the mRNA and protein levels for ColVI are elevated in the hippocampal neurons of Alzheimer's disease patients, as well as in a commonly used transgenic mouse model of Alzheimer's disease expressing mutant human amyloid precursor protein (APP; Box 1) (Cheng et al., 2009). In another study, expression of *Col6a1*, *Col6a2* and *Col6a3* genes was detected in murine primary hippocampal neurons and reported to increase following UV irradiation of the cultured cells (Cheng et al., 2011). The addition of soluble ColVI, but not of other collagens such as types I and IV, to the culture medium rescued UV-induced apoptosis and limited dendrite shrinkage by acting through the protein kinase B (PKB or Akt; AKT1) and c-Jun N-terminal kinase (JNK; MAPK8) pathways. These experiments confirmed that ColVI is functional in neuronal cells and indicated a neuroprotective role for this protein in the brain upon injury (Cheng et al., 2011). More recently, *Col6a3* mRNA expression was detected in neurons throughout the mouse adult brain, even in the absence of any kind of injury (Zech et al., 2015). Recent work by our group, aimed at investigating the physiological role for ColVI in the brain, also reported ColVI expression in primary hippocampal neurons, as well as in the meninges and brain blood vessels, in the hippocampal region, and in the corpus callosum (Box 1). Moreover, we demonstrated the presence of distinctive neurodegenerative traits in aged ColVI knockout (*Col6a1*^−/−^) mice (Box 2), pointing at a protective role for ColVI in physiological aging (Cescon et al., 2016), further corroborating the concept that ColVI protects neuronal cells against age-dependent neurodegeneration. Using wild-type and *Col6a1*^−/−^ mice, we found that the *COL6* genes were upregulated during aging and demonstrated that geriatric (23-month-old) ColVI-null mice displayed a higher incidence of apoptosis, higher oxidative damage and altered autophagic flux in neuronal cells compared with age-matched wild-type mice. The occurrence of neurodegenerative hallmarks in aged ColVI-null mice was accompanied by impaired motor coordination and spatial memory, highlighting a crucial neuroprotective role of ColVI during physiological aging (Cescon et al., 2016).

In the published studies aimed at identifying transcriptional profiles and signatures associated with neuronal network architecture, *COL6A1* was even listed among a selected number of genes involved in cortical lamination in humans (Krienen et al., 2016). *COL6A1* was also reported within a set of genes that are upregulated in the human cerebral cortex with respect to nonhuman primates, indicating a specific involvement of this gene in human brain development and evolution (Caceres et al., 2003). Together, the data discussed above demonstrate that neuronal cells are able to produce and secrete ColVI, and that ColVI secretion is functional, mainly exerting protection of neurons against stress.

## ColVI in the PNS

**Fig. 2.**
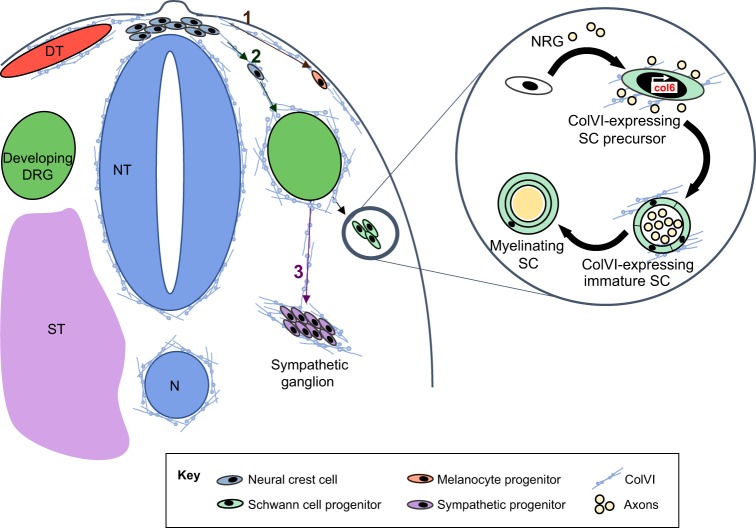
**ColVI is found along neural crest cell migratory pathways and regulates Schwann cell differentiation.** During embryonic development, neural crest cells (NCCs) originate from the neural tube closure site. After clustering at the dorsal apex of the neural tube (blue, NT), they soon migrate along two alternative paths: dorsolateral (1) or ventral (2) (Jessen and Mirsky, 2005). Along the dorsolateral path, ColVI is detectable in close proximity to the neural tube basement membrane, in the subectodermal basement membrane and surrounding the dermatome (red, DT) (Perris et al., 1993). NCCs entering this route give rise to melanocytes, once they colonize the developing dermis. At developmental stages following gangliogenesis, ColVI is found all around the developing dorsal root ganglia (green, DRG) and in the ventral sclerotome (purple, ST) (Perris et al., 1993). Indeed, NCCs can stop at the ventrolateral site of the developing DRG (2) or continue in a more ventral direction (3) towards the sclerotome, where they will give rise to the sympathetic ganglia (Jessen and Mirsky, 2005). The involvement of ColVI in Schwann cell (SC) differentiation is depicted in the enlarged circular inset. The immature Schwann cells acquire the competence to express ColVI via axon-derived neuregulin (NRG) signals, and soon after that, ColVI expression becomes NRG independent. Once SCs develop their mature myelinating phenotype, they cease to express *COL6* (Vitale et al., 2001).

Although the detailed dissection of ColVI expression and distribution in the CNS is still an open field of research, ColVI expression in the PNS is well known to be much more abundant, both during embryogenesis and in postnatal life. ColVI deposition in the PNS mostly occurs in the distal portion of the nerves, as a sheath surrounding axon bundles, and in the ventral and dorsal roots of spinal nerves, where weaker, but distinct, ColVI expression has been detected (Marvulli et al., 1996). The abundance of ColVI in the PNS allowed researchers to study it at a deeper level, leading to the dissection of its functions in this tissue from various developmental stages to adult age, and unraveling its mechanistic roles in the differentiation and regeneration of adult peripheral nerves (Fig. 2). During development, neural crest cells (NCCs; Box 1) that will give rise to Schwann cells (Box 1) and gangliar neurons in the PNS (Jessen and Mirsky, 2005) migrate out of the neural tube (Box 1) driven by the surrounding ECM, which actually guides them to reach their differentiation sites (Rogers et al., 1992). Immunolocalization studies in chick embryos detected ColVI in the ECM deposited on the dorsolateral side of the neural tube, corresponding to the primary migratory pathway of NCCs that will give rise to melanocytes in the skin. At later developmental stages, ColVI was also found in the ventral sclerotome (Box 1, Fig. 2) (Perris et al., 1993). Moreover, ColVI displays the ability to promote neural crest attachment and migration, thus supporting the concept that this ECM protein participates in the regulation of NCC movement by providing a suitable migratory substrate (Perris et al., 1993).

Studies on the transcriptional regulation of the murine *Col6a1* gene identified a specific enhancer region located ∼4.5 kb upstream of the TSS and driving its expression in the PNS (Girotto et al., 2000). A mechanistic study using transgenic *lacZ* reporter mouse lines demonstrated that ColVI expression in immature Schwann cells is initiated by neuregulin, a neuronal growth factor that is released by the growing nerve endings (Vitale et al., 2001). This occurs at the stage in which immature Schwann cells start differentiating into myelinating ones. The dependence on neuregulin is lost as soon as ColVI expression is elicited, and once Schwann cells acquire their mature myelinating phenotype, they cease to express ColVI (Fig. 2) (Vitale et al., 2001). Further studies confirmed the presence of ColVI in the adult connective tissue of endo-, peri- and epineurium (Box 1), deposited in the basement membrane by Schwann and perineural cells (Peltonen et al., 1990; Chen et al., 2014). Indeed, the proper assembly of the Schwann cell/neuron basement membrane was shown to be crucial in regulating Schwann cell maturation and myelination (Chernousov et al., 2008).

As reported for other ECM components, such as laminin-2 (Deodato et al., 2002; Yang et al., 2005), laminin-8 (Wallquist et al., 2005) and collagen XV (Rasi et al., 2010), ColVI displays a pivotal role in regulating peripheral nerve myelination and function. Using the ColVI knockout mouse model (Box 2), our group demonstrated that ColVI is necessary to ensure proper myelination. This was supported by *in vivo* evidence that a lack of ColVI in the *Col6a1*^−/−^ mouse model induces peripheral nerve hypermyelination in adult animals. The effects of ColVI on Schwann cells involve the activation of the signaling pathways that promote myelination, such as focal adhesion kinase (Fak; PTK2), Akt, extracellular signal-regulator kinase (Erk; MAPK1) and p38-mitogen activated protein kinase (p38; MAPK14), with a concomitant inhibition of negative regulators, including vimentin, JNK and Jun proto-oncogene (c-Jun; JUN) (Chen et al., 2014). Additionally, *in vitro* experiments showed that Schwann cells cultured on a ColVI substrate display decreased expression of myelin-associated proteins, further pointing at an inhibitory effect of ColVI on myelination (Chen et al., 2014). The structural alterations of myelin in peripheral nerves in ColVI knockout mice are associated with functional deficits, such as reduced nerve conduction velocity (Box 1) with shorter internodal length, and impaired motor coordination, as revealed by ledged-beam walking tests (Box 1) and footprint analyses. Sensory functions also appeared affected by ColVI ablation, because *Col6a1*^−/−^ mice had disorganized C-fibers and exhibited delayed nociception (Box 1) responses to thermal and mechanosensory stimulations (Chen et al., 2014).

The role of ColVI in peripheral nerve regeneration was also studied. Indeed, a recent study revealed that ColVI expression is rapidly increased at the site of sciatic crush injury in wild-type mice, and this event has a key role in the recruitment of macrophages and in their polarization towards the M2/pro-regenerative phenotype. The lack of ColVI in *Col6a1*^−/−^ mice causes delayed sciatic nerve regeneration upon injury, with significantly decreased macrophage recruitment to the site of injury and decreased M2 polarization when compared with similarly treated wild-type mice (Chen et al., 2015).

## ColVI involvement in CNS diseases

**Fig. 3.**
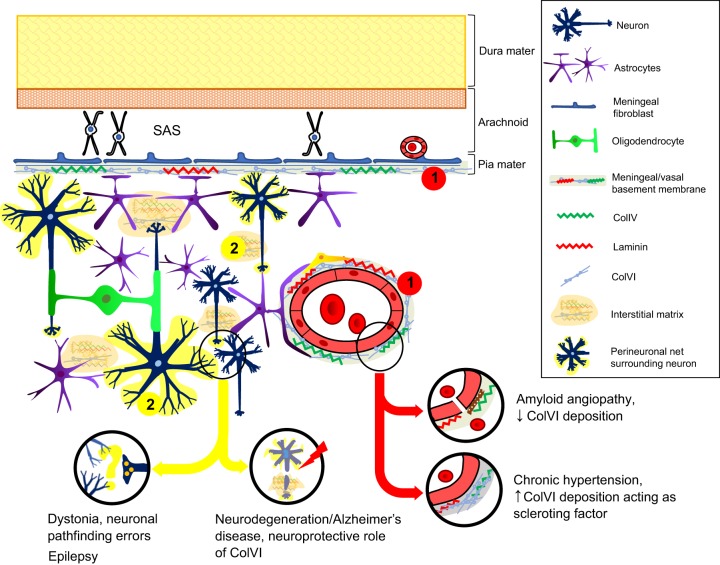
**Pathological aspects of the brain related to ColVI alterations.** In the brain, the ECM is associated with the meningeal and vessel basement membranes (red, 1) or forms perineuronal nets and the neural interstitial matrix (yellow, 2) (Kamei et al., 1992; Roggendorf et al., 1988; Zech et al., 2015). Alterations in ColVI deposition associated with the basement membrane were reported in amyloid angiopathy and chronic hypertension (Zhang et al., 1998; Roggendorf et al., 1988). Moreover, ColVI displays cytoprotective roles against neuronal cell death induced by stress (such as UV irradiation and amyloid-β toxicity) and aging (Cheng et al., 2009, 2011; Cescon et al., 2015). Mutations in ColVI were recently linked to defective neuronal pathfinding and altered synaptic plasticity, leading to dystonia and epilepsy, respectively (Zech et al., 2015; Karkheiran et al., 2013). SAS, subarachnoid space.

Although ColVI expression and deposition in the brain has not yet been completely characterized, a number of recently published studies link ColVI to neurological disorders, suggesting a crucial, though still poorly defined, role of this ECM component in CNS homeostasis (Fig. 3). ColVI was shown to be neuroprotective in the presence of cellular stress (Cheng et al., 2011), as well as in neurodegeneration. As discussed above, higher levels of the *Col6a1* transcript and protein were observed in hippocampal neurons of mice expressing mutant human APP, and a higher expression of *COL6A1*, *COL6A2* and *COL6A3* mRNA was also detected in the dentate gyrus (Box 1) of Alzheimer's disease patients compared with cognitively normal controls (Cheng et al., 2009). Treatment of murine primary neuron cultures with amyloid-β (Aβ)-42 peptides, the 42 kDa neurotoxic cleavage product of APP often found in Alzheimer's disease, stimulated ColVI expression via a TGF-β type II receptor-dependent mechanism. Moreover, ColVI treatment of primary neuronal cultures prevented the interaction of Aβ-42 oligomers with neurons, supporting a neuroprotective effect for ColVI (Cheng et al., 2009). The neuroprotective response elicited by ColVI does not appear to be exclusively directed against Aβ-42-dependent injury, since an independent study showed a protective effect for ColVI against UV-induced apoptosis via the activation of Akt/phosphatidylinositol 3-kinase (PI3K; PIK3CA) signaling (Cheng et al., 2011).

Notably, recent studies described the occurrence of *COL6* gene mutations in human CNS diseases (Fig. 1C). Compound heterozygous mutations of *COL6A3* were found in five patients suffering from autosomal recessive early-onset isolated dystonia (DYT27, OMIM #616411), a neurological disorder characterized by involuntary muscle contractions (Zech et al., 2015; Jochim et al., 2016). The mutations were identified in exons 36, 41 and 42 of *COL6A3*, encoding for the C1 (exon 36) and C4 (exons 41 and 42) domains at the C-terminal end of the α3(VI) chain, and affect amino acid residues that are highly conserved among mammalian species. *In situ* hybridization (Box 1) in adult mouse brain sections showed that neurons, including those located in the motor areas and known to be related to dystonia, express the *Col6a3* gene (Zech et al., 2015).

*In vivo* studies in zebrafish (Box 2), in which the ortholog of human *COL6A3* exon 41 was knocked down by morpholino oligonucleotides (Box 1), revealed abnormalities in motor neuron pathfinding and defective neuronal branching and extension (Fig. 3) (Zech et al., 2015). These findings suggest that mutations of the fibronectin III (FN-III) motif of the C4 domain of the α3(VI) chain (Fig. 1A) might affect the correct early development of neuron circuits, as well as the synaptic remodeling of adult brain. This is in agreement with the finding that the FN-III motif of another ECM protein, tenascin-C, is able to modulate synaptic plasticity and axonal outgrowth (Strekalova et al., 2002), thus suggesting a role for the α3(VI) C4 domain in synaptogenesis and in the maintenance and/or stability of neural circuits.

Mutations of the *COL6A2* gene were linked to another neurodegenerative disease, progressive myoclonus epilepsy syndrome (PME), characterized by seizures, myoclonus, ataxia, cognitive defects and early death (Karkheiran et al., 2013). This study described a consanguineous family in which two siblings diagnosed with PME carried a homozygous missense mutation in the *COL6A2* gene, causing an Asp to Asn substitution in the vWFA module of the N-terminal region of the α2(VI) chain. Interestingly, chromosomal aberrations involving the *COL6A2* gene were previously linked to febrile seizures and idiopathic generalized epilepsy (Kim et al., 2007). These findings are of great interest, because the relevance of collagens and ECM remodeling in the onset of epilepsies is widely accepted (Dityatev and Fellin, 2008), thus highlighting a fundamental role of the ECM in brain physiology and pathology.

Differently, the contribution of ColVI to brain vessel architecture can be appreciated in pathological states involving ECM remodeling, such as cerebrovascular diseases (Fig. 3). Changes in the distribution of different collagen types in brain vessels were studied in postmortem biopsies from patients affected by amyloid angiopathy (Box 1) (Zhang et al., 1998). In these biopsies, the tunica media of amyloid-containing vessels exhibited a strong decrease in collagen types I, III, V and VI compared to control biopsies. These findings were remarkably different to what was seen in other angiopathies, where increased amounts of collagens are deposited in the ECM (Zhang and Olsson, 1997), and the authors suggested that the higher rate of vessel rupture seen in amyloid angiopathy might be caused by the degeneration of the tunica media associated with the decreased collagen deposition in the ECM.

ColVI deposition was also investigated in brain samples from patients affected by chronic hypertension, and compared with normal brains (Roggendorf et al., 1988). In the controls, ColVI was detected in the adventitia of meningeal and larger intraparenchymal vessels, as well as in the choroid plexus and glia limitans. ColVI deposition in hypertensive patients extended into the vascular intima and media, and in cortical vessels, probably acting as a sclerosing component during hypertension. In a number of independent studies, ColVI was associated with glaucoma, a central optic neuropathy. The optic nerve is considered part of the CNS, as it derives from an outpocketing of the diencephalon (Box 1) during embryonic development (Guillery et al., 1995). Besides their optic nerve origin, glaucomatous injuries are increasingly associated with classical neurodegenerative diseases owing to some common features, such as neuronal loss (Yu et al., 2013). Glaucoma is caused by high intraocular pressure, which leads to retinal ganglion cell death, optic axon degeneration and blindness (Weinreb et al., 2014). Primary open-angle glaucoma, one of the most frequent forms, is also characterized by elastosis of the prelaminar and laminar regions, and increased connective tissue deposition in the postlaminar region (Hernandez et al., 1990). An increased deposition of ColVI was reported in glaucoma (Johnson et al., 2007) and it was suggested to be induced in optic nerve astrocytes by growth factors, such as TGF-β2, circulating in the aqueous humor (Box 1) (Neumann et al., 2008). Indeed, treatment of cultured human primary astrocytes with TGF-β2 induces the expression of connective tissue growth factor which, in turn, upregulates ColVI, together with elastin (Neumann et al., 2008).

As evident from the studies described above, CNS diseases linked to ColVI span a remarkable range of tissues and compartments, from the hippocampus to the optic nerve, and from neurons to brain vessels, thus pointing at a broad implication for ColVI in the proper maintenance of CNS homeostasis.

## ColVI involvement in PNS diseases

Besides the increasing number of CNS diseases linked to ColVI genes, a much deeper characterization of ColVI localization and function was achieved in the PNS. However, a precise association of this ECM component with myelinating diseases or neuropathies, as it may be expected according to phenotypic studies in mice, has not yet been obtained. In spite of this, abnormal ColVI expression in pathological states affecting the PNS was reported by different groups.

An interesting case is represented by Hirschsprung disease (HSCR), a congenital affection of the enteric nervous system characterized by deficient innervation of the distal bowel, thus leading to severe functional obstructions (Amiel et al., 2007). A recent study described the genetic and phenotypic characterization of a novel animal model for HSCR, called the Holstein mouse (*Hol^Tg/Tg^*). This line was generated through an insertional mutagenesis screening (Box 1) for genes important for NCC development. The molecular analysis of *Hol^Tg/Tg^* mice identified an untargeted transgenic insertion upstream of the *Col6a4* gene, causing upregulation of ColVI expression in enteric NCCs (eNCCs). Phenotypically, these animals die prematurely due to aganglionic megacolon (Box 1) (Soret et al., 2015). The increased ColVI synthesis and ECM deposition by eNCCs during development is supposed to affect their migration capabilities, with defective eNCC migration in *Hol^Tg/Tg^* embryos. Additional studies demonstrated a less efficient response of eNCCs to the migration-promoting effects of fibronectin in *ex vivo* midgut explants (Heuckeroth, 2015). Remarkably, an increased ECM deposition of ColVI was found around the enteric ganglia of HSCR patients (Soret et al., 2015). By emphasizing that Down syndrome patients are strongly predisposed to HSRC, Heuckenroth and colleagues proposed that the *COL6A1/2* genes, located on human Chr.21q and overexpressed in trisomy 21 fetuses (Von Kaisenberg et al., 1998) and postnatal intestines (Soret et al., 2015), could also be candidates for predisposition to HSCR (Heuckeroth, 2015).

ColVI has been associated with diabetic peripheral neuropathy, a long-term complication of the primary disease and characterized by distal axonopathy, loss of nerve fibers and Wallerian degeneration (Box 1) (Yagihashi et al., 2007). Remodeling of the ECM occurs as a consequence of diabetes mellitus and is supposed to influence the nerve regeneration processes (Peltonen et al., 1997; Hill, 2009). Abnormal deposition of ColVI in human diabetic neuropathy was reported by Muona and colleagues, who observed a thickening of the perineural basement membranes, together with the presence of ColVI-positive microfibrils and Luse bodies (Box 1) (Muona et al., 1993). In a spontaneous diabetic rat model, the authors corroborated the presence of ColVI-positive microfibrils in the neuropathic perineural basement membrane. Moreover, they demonstrated that ColVI expression was upregulated *in vitro* upon glucose treatment of co-cultured perineural cells, Schwann cells and neural fibroblasts, thus indicating altered ColVI biosynthesis due to the hyperglycemic environment (Muona et al., 1993). Consistent with these findings, the deposition of ColVI, as well as of tenascin-C and collagen V, was significantly elevated in endoneurial connective tissues of patients affected by diabetic neuropathy (Hill, 2009). A significant increase in ColVI deposition was found in the perineurium of neuropathic nerves too, thus demonstrating broader changes to the connective tissue compartment. However, it remains to be established whether this ECM remodeling is a pro-regenerative response to diabetic nerve injury or, conversely, is a side effect of chronic nerve damage (Hill, 2009).

A reorganization of the ECM was also reported for hereditary neuropathies, such as Charcot-Marie-Tooth disease type I (CMT1), a demyelinating neuropathy caused by mutations of different genes expressed by myelinating Schwann cells. In healthy sural nerves, ColVI is present at the level of perineural layers, Schwann cells, epi- and endoneurial vessels and stroma, together with epineurial fibroblasts. In CMT1 patients, ColVI expression was found to be particularly increased in association with Schwann cells around myelinated fibers. A similar upregulation was also found in samples from patients affected by chronic idiopathic axonal polyneuropathy, another hereditary neuropathy (Palumbo et al., 2002).

Only recently, the first pieces of evidence concerning the link between mutations in *COL6* genes and peripheral neuropathic conditions were collected (Fig. 1C). Hunter and colleagues described the clinical features of two childhood myopathy cases whose electromyography (EMG; Box 1) revealed abnormalities suggestive of neuropathy or neurogenic defects. The candidate genetic variants were identified through whole-exome sequencing (Hunter et al., 2015). One of the two patients carried a homozygous missense mutation in *COL6A3*, which was already known to be pathogenic and linked to BM. Before a detailed assessment, this patient was erroneously assigned different diagnoses, including CMT, and EMG analysis suggested chronic motor neuropathy. A second patient, bearing the same mutation in one allele of *COL6A3* and a different variant affecting the second allele of the same gene, presented fibrillations and sharp waves in the anterior tibialis by EMG analysis, associated with evidence of muscle fiber splitting and muscle fiber atrophy (Hunter et al., 2015). Although defects that can imply neurogenic aspects, such as reduced nerve conduction velocity, were rarely reported in previous studies of patients affected by ColVI-related myopathies (namely, BM, UCMD and myosclerosis), these more recent findings suggest that more specific EMG analyses could help with better defining the previously unrecognized contributions of peripheral nerve defects to ColVI-related myopathies. Indeed, a retrospective study on EMG and nerve conduction in children affected by different congenital muscular dystrophies reported borderline reduced motor and sensory nerve conduction velocities in two out of three UCMD patients. These findings suggest that the pathogenesis and course of nerve involvement in ColVI-related congenital muscular dystrophies should be further analyzed (Quijano-Roy et al., 2004), because a potential contribution from the nervous compartment could be underestimated. In keeping with the challenges in discriminating between peripheral nerve and muscle alterations due to a degree of symptom overlap, a case was described of a woman diagnosed with distal hereditary motor neuropathy on the basis of clinical and EMG data. This patient carried a heterozygous variant of the *TRPV4* gene, a polymorphism reported in the healthy population, and a new heterozygous mutation in *COL6A1* (Leonard-Louis et al., 2016). The patient's genotype implies that the presence of polymorphisms and variants in other genes might differentially affect the clinical phenotype of patients with ColVI-related disorders.

Beside the above considerations about the potential presence of peripheral nerve defects in ColVI-related myopathies, a separate whole-exome sequencing study identified two rare *COL6A5* variants in three different families affected by neuropathic chronic itch, and characterized by reduced intradermal nerve fiber density. Immunofluorescence and western blot analyses revealed markedly reduced α5(VI) expression (Martinelli-Boneschi et al., 2017). This recent study represents the first report of peripheal neuropathy specifically associated with *COL6* mutations in humans. Interestingly, tissue-specific expression of the different ColVI chains was previously reported (Gara et al., 2008, 2011). The identification of specific *COL6* mutations linked to a major nerve defect points at the potential involvement of unique ColVI domains as well as of distinct receptors.

## ColVI in benign and malignant tumors of the nervous system

ColVI has been implicated in benign and malignant tumors affecting various tissues, spanning from colon to breast, ovary, lung and skin. ColVI exerts various pro-tumorigenic effects, such as by acting on the Akt–glycogen synthase kinase (GSK) 3β–β-catenin–T cell factor/lymphoid enhancer factor (TCF/LEF) axis, enhancing the production of pro-tumorigenic factors, and increasing the recruitment of tumor-associated macrophages (Chen et al., 2013).

The initial studies reporting ColVI expression in nervous system tumors were conducted in cultured glioblastoma cell lines. ColVI deposition in the cultures was postulated to be actively involved in tumor invasiveness, because glioblastoma cells cultured on rat brain slices displayed strong ColVI labeling in the tumor mass and in the invading cells (Han et al., 1994, 1995). In agreement with this, recent studies associated ColVI with glioma, the most common primary tumor of the CNS. Gliomas are classified in four grades based on their histological and malignant features, from the lowest, more benign pilocytic astrocytoma (grade I), to the highest, more aggressive glioblastoma multiforme (grade IV) (Louis et al., 2007). Serial analysis of gene expression (SAGE) of gliomas of different grades and of nonmalignant CNS specimens showed differential expression of *COL6A1* in the different tumor grades, with the highest expression levels detected in aggressive grade III and IV glioblastoma, compared with a reduced expression in lower-grade (grade I and II) astrocytoma and normal glia (Fujita et al., 2008). Although this indicates that *COL6A1* expression might be a diagnostic marker for tumor progression, as its increased expression is a hallmark of high-grade gliomas, further studies pointed at *COL6A1* as a prognostic marker, because it is also associated with poor clinical outcome in grade IV glioblastoma multiforme (Turtoi et al., 2014). Additionally, *COL6A2* expression was also reported to be associated with high-grade astrocytomas (Boon et al., 2004), and gene expression profiling showed that both *COL6A2* and *COL6A3* are upregulated in pediatric brain tumors, including pilocytic astrocytoma, ependymoma, medulloblastoma and glioblastoma multiforme (Di Rosa et al., 2015).

In agreement with earlier work identifying ColVI co-distribution with fibronectin in the vasculature and stromal connective tissue of human gliomas (McComb et al., 1987), further studies demonstrated the association of ColVI with the basal lamina of brain tumor vessels (Huang et al., 2010; You et al., 2012). Intracranial injection of B16F10 melanoma tumor cells into the corpus callosum of wild-type and *Col6a1*^−/−^ mice demonstrated that brain tumors are not able to grow in the absence of ColVI, owing to an impairment of vascular basal lamina organization (You et al., 2012). Indeed, the lack of ColVI caused a reduced deposition of the basal lamina in tumor vessels, which was associated with delayed pericyte maturation and endothelial cell apoptosis. Ultimately, these abnormalities contributed to a reduced patency and an increased leakiness of tumor vessels (You et al., 2012), thus revealing a crucial role for ColVI in brain tumor angiogenesis.

Consistent with the described ColVI deposition in the meninges, tumors affecting these specialized tissue layers are characterized by distinctive ColVI labeling. ColVI has been detected on the surface of the psammoma (Box 1) bodies of human meningiomas, as well as on the tumor cells and in the surrounding interstitial matrix (Han et al., 1996). Clear cell meningioma, characterized by the presence of malignant cells filled with abundant glycogen (the so-called clear cells), frequently presents with ECM deposits that are strongly positive for ColVI and are considered to represent the degenerative remnants of long-lasting persistent tumors (Kubota et al., 1995; Payano et al., 2004). Moreover, like for gliomas, ColVI chains were found to be differentially expressed in meningiomas, depending on tumor grade. In particular, proteomic analyses showed increased α1(VI) levels in grade I meningiomas, as well as increased α3(VI) levels in benign and low-grade meningiomas compared with higher-grade ones (Sharma et al., 2015).

The involvement of ColVI was also demonstrated in tumors affecting the peripheral nervous tissue. Tumors originating in the PNS are characterized by complex histological features, owing to the composite structure of the tissue itself, which consists of distinct cell types of different developmental origins. For example, neurofibromas and schwannomas derive from Schwann cells, whereas tumors arising from neurons give origin to neuromas (Ariel, 1988). Oda and colleagues reported that collagen staining in schwannomas displays a peculiar distribution, depending on specific tissue areas and cellular patterns. In particular, strong ColVI labeling was detected in bundles of microfibrils present in the intracellular space of the so-called Antoni type B regions of schwannomas (Oda et al., 1988). In another study, a combination of *in situ* hybridization and immunohistochemistry demonstrated that the majority of neurofibroma cells express high levels of *COL6A2* and its protein product (Peltonen et al., 1990). Finally, a study focusing on the distribution of collagen gene expression in cutaneous neurofibromas in relation to blood vessels showed that distinct subpopulations of endothelial cells express high levels of *COL6* mRNAs, suggesting that ColVI deposition might contribute to the growth and architecture of neurofibromas (Sollberg et al., 1991).

The literature studies published so far highlight the presence of ColVI in tumors of the nervous tissue, as was reported for other compartments (Chen et al., 2013). Based on these findings, some of the studies described above support differential ColVI expression as a promising diagnostic and prognostic factor. However, our knowledge concerning the active role of this ECM protein in the nervous system tumor microenvironment is still limited.

## Conclusions

Although a growing number of studies are increasingly highlighting the distinct roles of ColVI in the nervous system, much work remains to be done to reach a more detailed understanding of the functions of this protein in the CNS and PNS. In particular, the CNS represents a major gap in knowledge, reflecting this organ system's intrinsic complexity. This is becoming extraordinarily amplified, rather than deconstructed, by the increasing amount of data coming from genetic, transcriptomic and proteomic studies. Neuroscience, more than any other research field, requires appropriate animal models for the *in vivo* dissection of the multiple functional aspects of cell circuits and of the different factors affecting them. In this respect, the currently available, as well as novel *in vivo* models for investigating ColVI (Box 2) in the nervous system are of high value and represent a peerless resource. Although our knowledge of ColVI functions in the CNS and in the PNS is still partial, it is evident that the expression and deposition of this distinctive ECM component in the nervous system is highly dynamic and tightly controlled. It is finely tuned in different regions of the brain and in peripheral nerves, and across various developmental stages, but it is also regulated in response to different stress conditions. *COL6* genes have already been linked to different pathological conditions of the brain. The available data suggest that neurodevelopment could become a major field of future ColVI investigation, especially to identify further molecular and genetic defects of ColVI that are involved in disease phenotypes affecting the nervous system.

## References

[DMM032946C1] Amiel J., Sproat-Emison E., Garcia-Barcelo M., Lantieri F., Burzynski G., Borrego S., Pelet A., Arnold S., Miao X., Griseri P. et al. (2007). Hirschsprung disease, associated syndromes and genetics: a review. *J. Med. Genet.* 45, 1-14. 10.1136/jmg.2007.053959 17965226

[DMM032946C2] Ariel I. M. (1988). Tumors of the peripheral nervous system. *Semin. Surg. Oncol.* 4, 7-12. 10.1002/ssu.2980040104 3127871

[DMM032946C3] Bonaldo P. and Colombatti A. (1989). The carboxyl terminus of the chicken α3 chain of collagen VI is a unique mosaic structure with glycoprotein Ib-like, fibronectin type III, and Kunitz modules. *J. Biol. Chem.* 264, 20235-20239. 2584214

[DMM032946C4] Bonaldo P., Russo V., Bucciotti F., Bressan G. M. and Colombatti A. (1989). α1 chain of chick type VI collagen. The complete cDNA sequence reveals a hybrid molecule made of one short collagen and three von Willebrand Factor type A-like domains. *J. Biol. Chem.* 264, 5575-5580. 2784434

[DMM032946C5] Bonaldo P., Russo V., Bucciotti F., Doliana R. and Colombatti A. (1990). Structural and functional features of the alpha 3 chain indicate a bridging role for chicken collagen VI in connective tissues. *Biochemistry* 29, 1245-1254. 10.1021/bi00457a021 2322559

[DMM032946C6] Bonaldo P., Braghetta P., Zanetti M., Piccolo S., Volpin D. and Bressan G. M. (1998). Collagen VI deficiency induces early onset myopathy in the mouse: an animal model for Bethlem myopathy. *Hum. Mol. Genet.* 7, 2135-2140. 10.1093/hmg/7.13.2135 9817932

[DMM032946C7] Bönnemann C. G. (2011). The collagen VI-related myopathies: muscle meets its matrix. *Nat. Rev. Neurol.* 7, 379-390. 10.1038/nrneurol.2011.81 21691338 PMC5210181

[DMM032946C8] Boon K., Edwards J. B., Eberhart C. G. and Riggins G. J. (2004). Identification of astrocytoma associated genes including cell surface markers. *BMC Cancer* 4, 39 10.1186/1471-2407-4-39 15265232 PMC497045

[DMM032946C9] Braghetta P., Fabbro C., Piccolo S., Marvulli D., Bonaldo P., Volpin D. and Bressan G. M. (1996). Distinct regions control transcriptional activation of the α1(VI) collagen promoter in different tissues of transgenic mice. *J. Cell Biol.* 135, 1163-1177. 10.1083/jcb.135.4.1163 8922394 PMC2133380

[DMM032946C10] Bruns R. R., Press W., Engvall E., Timpl R. and Gross J. (1986). Type VI collagen in extracellular, 100-nm periodic filaments and fibrils: identification by immunoelectron microscopy. *J. Cell Biol.* 103, 393-404. 10.1083/jcb.103.2.393 3525575 PMC2113834

[DMM032946C11] Caceres M., Lachuer J., Zapala M. A., Redmond J. C., Kudo L., Geschwind D. H., Lockhart D. J., Preuss T. M. and Barlow C. (2003). Elevated gene expression levels distinguish human from non-human primate brains. *Proc. Natl. Acad. Sci. USA* 100, 13030-13035. 10.1073/pnas.2135499100 14557539 PMC240739

[DMM032946C12] Cescon M., Gattazzo F., Chen P. and Bonaldo P. (2015). Collagen VI at a glance. *J. Cell Sci.* 128, 3525-3531. 10.1242/jcs.169748 26377767

[DMM032946C13] Cescon M., Chen P., Castagnaro S., Gregorio I. and Bonaldo P. (2016). Lack of collagen VI promotes neurodegeneration by impairing autophagy and inducing apoptosis during aging. *Aging* 8, 1083-1101. 10.18632/aging.100924 27060109 PMC4931855

[DMM032946C14] Chen P., Cescon M. and Bonaldo P. (2013). Collagen VI in cancer and its biological mechanisms. *Trends Mol. Med.* 19, 410-417. 10.1016/j.molmed.2013.04.001 23639582

[DMM032946C15] Chen P., Cescon M., Megighian A. and Bonaldo P. (2014). Collagen VI regulates peripheral nerve myelination and function. *FASEB J.* 28, 1145-1156. 10.1096/fj.13-239533 24277578

[DMM032946C16] Chen P., Cescon M., Zuccolotto G., Nobbio L., Colombelli C., Filaferro M., Vitale G., Feltri M. L. and Bonaldo P. (2015). Collagen VI regulates peripheral nerve regeneration by modulating macrophage recruitment and polarization. *Acta Neuropathol.* 129, 97-113. 10.1007/s00401-014-1369-9 25421425

[DMM032946C17] Cheng J. S., Dubal D. B., Kim D. H., Legleiter J., Cheng I. H., Yu G.-Q., Tesseur I., Wyss-Coray T., Bonaldo P. and Mucke L. (2009). Collagen VI protects neurons against Aβ toxicity. *Nat. Neurosci.* 12, 119-121. 10.1038/nn.2240 19122666 PMC2812922

[DMM032946C18] Cheng I. H., Lin Y.-C., Hwang E., Huang H.-T., Chang W.-H., Liu Y.-L. and Chao C.-Y. (2011). Collagen VI protects against neuronal apoptosis elicited by ultraviolet irradiation via an Akt/Phosphatidylinositol 3-kinase signaling pathway. *Neuroscience* 183, 178-188. 10.1016/j.neuroscience.2011.03.057 21459131

[DMM032946C19] Chernousov M. A., Yu W.-M., Chen Z.-L., Carey D. J. and Strickland S. (2008). Regulation of Schwann cell function by the extracellular matrix. *Glia* 56, 1498-1507. 10.1002/glia.20740 18803319

[DMM032946C20] Chu M.-L., Mann K., Deutzmann R., Pribula-Conway D., Hsu-Chen C.-C., Bernard M. P. and Timpl R. (1987). Characterization of three constituent chains of collagen type VI by peptide sequences and cDNA clones. *Eur. J. Biochem.* 168, 309-317. 10.1111/j.1432-1033.1987.tb13422.x 3665927

[DMM032946C21] Chu M. L., Conway D., Pan T. C., Baldwin C., Mann K., Deutzmann R. and Timpl R. (1988). Amino acid sequence of the triple-helical domain of human collagen type VI. *J. Biol. Chem.* 263, 18601-18606. 3198591

[DMM032946C22] Chu M. L., Zhang R. Z., Pan T. C., Stokes D., Conway D., Kuo H. J., Glanville R., Mayer U., Mann K., Deutzmann R. et al. (1990a). Mosaic structure of globular domains in the human type VI collagen alpha3 chain: similarity to von Willebrand factor, fibronectin, actin, salivary proteins and aprotinin type protease inhibitors. *EMBO J.* 9, 385-393. 1689238 10.1002/j.1460-2075.1990.tb08122.x PMC551678

[DMM032946C23] Chu M.-L., Pan T.-C., Conway D., Saitta B., Stokes D., Kuo H.-J., Glanville R. W., Timpl R., Mann K. and Deutzmann R. (1990b). The structure of type VI collagen. *Ann. NY Acad. Sci.* 580, 55-63. 10.1111/j.1749-6632.1990.tb17917.x 2337306

[DMM032946C24] Colombatti A. and Bonaldo P. (1987). Biosynthesis of chick type VI collagen II. Processing and secretion in fibroblasts and smooth muscle cells. *J. Biol. Chem.* 262, 14461-14466. 3667584

[DMM032946C25] Colombatti A., Bonaldo P., Ainger K., Bressan G. M. and Volpin D. (1987). Biosynthesis of chick type VI collagen. I. Intracellular assembly and molecular structure. *J. Biol. Chem.* 262, 14454-14460. 3117786

[DMM032946C26] Colombatti A., Mucignat M. T. and Bonaldo P. (1995). Secretion and matrix assembly of recombinant type VI collagen. *J. Biol. Chem.* 270, 13105-13111. 10.1074/jbc.270.22.13105 7768905

[DMM032946C27] Deodato F., Sabatelli M., Ricci E., Mercuri E., Muntoni F., Sewry C., Naom I., Tonali P. and Guzzetta F. (2002). Hypermyelinating neuropathy, mental retardation and epilepsy in a case of merosin deficiency. *Neuromuscul. Disord.* 12, 392-398. 10.1016/S0960-8966(01)00312-1 12062258

[DMM032946C28] Di Rosa M., Sanfilippo C., Libra M., Musumeci G. and Malaguarnera L. (2015). Different pediatric brain tumors are associated with different gene expression profiling. *Acta Histochem.* 117, 477-485. 10.1016/j.acthis.2015.02.010 25792036

[DMM032946C29] Dityatev A. and Fellin T. (2008). Extracellular matrix in plasticity and epileptogenesis. *Neuron Glia Biol.* 4, 235-247. 10.1017/S1740925X09000118 19497143

[DMM032946C30] Dziadek M., Darling P., Bakker M., Overall M., Zhang R.-Z., Pan T.-C., Tillet E., Timpl R. and Chu M.-L. (1996). Deposition of collagen VI in the extracellular matrix during mouse embryogenesis correlates with expression of the α3(VI) subunit gene. *Exp. Cell Res.* 226, 302-315. 10.1006/excr.1996.0231 8806434

[DMM032946C31] Egan R. J., Bergner C. L., Hart P. C., Cachat J. M., Canavello P. R., Elegante M. F., Elkhayat S. I., Bartels B. K., Tien A. K., Tien D. H. et al. (2009). Understanding behavioral and physiological phenotypes of stress and anxiety in zebrafish. *Behav. Brain Res.* 205, 38-44. 10.1016/j.bbr.2009.06.022 19540270 PMC2922906

[DMM032946C32] Engvall E., Hessle H. and Klier G. (1986). Molecular assembly, secretion, and matrix deposition of type VI collagen. *J. Cell Biol.* 102, 703-710. 10.1083/jcb.102.3.703 3456350 PMC2114116

[DMM032946C33] Fitzgerald J., Rich C., Zhou F. H. and Hansen U. (2008). Three novel collagen VI chains, α4(VI), α5(VI), and α6(VI). *J. Biol. Chem.* 283, 20170-20180. 10.1074/jbc.M710139200 18400749

[DMM032946C34] Fujita A., Sato J. R., Festa F., Gomes L. R., Oba-Shinjo S. M., Marie S. K. N., Ferreira C. E. and Sogayar M. C. (2008). Identification of COL6A1 as a differentially expressed gene in human astrocytomas. *Genet. Mol. Res.* 7, 371-378. 10.4238/vol7-2gmr432 18551403

[DMM032946C35] Gara S. K., Grumati P., Urciuolo A., Bonaldo P., Kobbe B., Koch M., Paulsson M. and Wagener R. (2008). Three novel collagen VI chains with high homology to the α3 chain. *J. Biol. Chem.* 283, 10658-10670. 10.1074/jbc.M709540200 18276594

[DMM032946C36] Gara S. K., Grumati P., Squarzoni S., Sabatelli P., Urciuolo A., Bonaldo P., Paulsson M. and Wagener R. (2011). Differential and restricted expression of novel collagen VI chains in mouse. *Matrix Biol.* 30, 248-257. 10.1016/j.matbio.2011.03.006 21477648

[DMM032946C37] Girotto D., Fabbro C., Braghetta P., Vitale P., Volpin D. and Bressan G. M. (2000). Analysis of transcription of the Col6a1 gene in a specific set of tissues suggests a new variant of enhancer region. *J. Biol. Chem.* 275, 17381-17390. 10.1074/jbc.M000075200 10747869

[DMM032946C38] Guillery R. W., Mason C. A. and Taylor J. S. (1995). Developmental determinants at the mammalian optic chiasm. *J. Neurosci.* 15, 4727-4737. 10.1523/JNEUROSCI.15-07-04727.1995 7623106 PMC6577905

[DMM032946C39] Han J., Daniel J. C., Lieska N. and Pappas G. D. (1994). Immunofluorescence and biochemical studies of the type VI collagen expression by human glioblastoma cells in vitro. *Neurol. Res.* 16, 370-375. 10.1080/01616412.1994.11740256 7870276

[DMM032946C40] Han J., Daniel J. C. and Pappas G. D. (1995). Expression of type VI collagen during glioblastoma cell invasion in brain tissue cultures. *Cancer Lett.* 88, 127-132. 10.1016/0304-3835(94)03627-U 7874684

[DMM032946C41] Han J., Daniel J. C. and Pappas G. D. (1996). Expression of type VI collagen in psammoma bodies: immunofluorescence studies on two fresh human meningiomas. *Acta Cytol.* 40, 177-181. 10.1159/000333688 8629394

[DMM032946C42] Hernandez M. R., Andrzejewska W. M. and Neufeld A. H. (1990). Changes in the extracellular matrix of the human optic nerve head in primary open-angle glaucoma. *Am. J. Ophthalmol.* 109, 180-188. 10.1016/S0002-9394(14)75984-7 2405683

[DMM032946C43] Heuckeroth R. O. (2015). Hirschsprung's disease, Down syndrome, and missing heritability: too much collagen slows migration. *J. Clin. Invest.* 125, 4323-4326. 10.1172/JCI85003 26571392 PMC4665790

[DMM032946C44] Hill R. (2009). Extracellular matrix remodelling in human diabetic neuropathy. *J. Anat.* 214, 219-225. 10.1111/j.1469-7580.2008.01026.x 19207983 PMC2667879

[DMM032946C45] Huang F.-J., You W.-K., Bonaldo P., Seyfried T. N., Pasquale E. B. and Stallcup W. B. (2010). Pericyte deficiencies lead to aberrant tumor vascularization in the brain of the NG2 null mouse. *Dev. Biol.* 344, 1035-1046. 10.1016/j.ydbio.2010.06.023 20599895 PMC3197744

[DMM032946C46] Hunter J. M., Ahearn M. E., Balak C. D., Liang W. S., Kurdoglu A., Corneveaux J. J., Russell M., Huentelman M. J., Craig D. W., Carpten J. et al. (2015). Novel pathogenic variants and genes for myopathies identified by whole exome sequencing. *Mol. Genet. Genomic Med.* 3, 283-301. 10.1002/mgg3.142 26247046 PMC4521965

[DMM032946C47] Jessen K. R. and Mirsky R. (2005). The origin and development of glial cells in peripheral nerves. *Nat. Rev. Neurosci.* 6, 671-682. 10.1038/nrn1746 16136171

[DMM032946C48] Jochim A., Zech M., Gora-Stahlberg G., Winkelmann J. and Haslinger B. (2016). The clinical phenotype of early-onset isolated dystonia caused by recessive COL6A3 mutations (DYT27). *Mov. Disord.* 31, 747-750. 10.1002/mds.26501 26687111

[DMM032946C49] Johnson E. C., Jia L., Cepurna W. O., Doser T. A. and Morrison J. C. (2007). Global changes in optic nerve head gene expression after exposure to elevated intraocular pressure in a rat glaucoma model. *Invest. Ophthalmol. Vis. Sci.* 48, 3161-3177. 10.1167/iovs.06-1282 17591886 PMC1950563

[DMM032946C50] Kamei A., Houdou S., Mito T., Konomi H. and Takashima S. (1992). Developmental change in type VI collagen in human cerebral vessels. *Pediatr. Neurol.* 8, 183-186. 10.1016/0887-8994(92)90065-7 1622513

[DMM032946C51] Karkheiran S., Krebs C. E., Makarov V., Nilipour Y., Hubert B., Darvish H., Frucht S., Shahidi G. A., Buxbaum J. D. and Paisán-Ruiz C. (2013). Identification of COL6A2 mutations in progressive myoclonus epilepsy syndrome. *Hum. Genet.* 132, 275-283. 10.1007/s00439-012-1248-1 23138527

[DMM032946C52] Keene D. R., Engvall E. and Glanville R. W. (1988). Ultrastructure of type VI collagen in human skin and cartilage suggests an anchoring function for this filamentous network. *J. Cell. Biol.* 107, 1995-2006. 10.1083/jcb.107.5.1995 3182942 PMC2115316

[DMM032946C53] Kim H. S., Yim S.-V., Jung K. H., Zheng L. T., Kim Y.-H., Lee K.-H., Chung S.-Y. and Rha H. K. (2007). Altered DNA copy number in patients with different seizure disorder type: by array-CGH. *Brain Dev.* 29, 639-643. 10.1016/j.braindev.2007.04.006 17573221

[DMM032946C54] Krienen F. M., Yeo B. T. T., Ge T., Buckner R. L. and Sherwood C. C. (2016). Transcriptional profiles of supragranular-enriched genes associate with corticocortical network architecture in the human brain. *Proc. Natl. Acad. Sci. USA* 113, E469-E478. 10.1073/pnas.1510903113 26739559 PMC4739529

[DMM032946C55] Kubota T., Sato K., Kabuto M., Hasegawa M., Kitai R., Nakagawa T., Arai Y. and Yamashita J. (1995). Clear cell (glycogen-rich) meningioma with special reference to spherical collagen deposits. *Noshuyo Byori* 12, 53-60. 7795730

[DMM032946C56] Lampe A. K. and Bushby K. M. D. (2005). Collagen VI related muscle disorders. *J. Med. Genet.* 42, 673-685. 10.1136/jmg.2002.002311 16141002 PMC1736127

[DMM032946C57] Lau L. W., Cua R., Keough M. B., Haylock-Jacobs S. and Yong V. W. (2013). Pathophysiology of the brain extracellular matrix: a new target for remyelination. *Nat. Rev. Neurosci.* 14, 722-729. 10.1038/nrn3550 23985834

[DMM032946C58] Leonard-Louis S., Latour P., De Becdelievre A., Themar-Noel C., Fournier E. and Stojkovic T. (2016). TRPV4 gene polymorphism as a phenotype modifier in a family with COL6-linked Bethlem myopathy. *Neuromuscul. Disord.* 26, S188 10.1016/j.nmd.2016.06.369

[DMM032946C59] Louis D. N., Ohgaki H., Wiestler O. D., Cavenee W. K., Burger P. C., Jouvet A., Scheithauer B. W. and Kleihues P. (2007). The 2007 WHO classification of tumours of the central nervous system. *Acta Neuropathol.* 114, 97-109. 10.1007/s00401-007-0243-4 17618441 PMC1929165

[DMM032946C60] Martinelli-Boneschi F., Colombi M., Castori M., Devigili G., Eleopra R., Malik R. A., Ritelli M., Zoppi N., Dordoni C., Sorosina M. et al. (2017). COL6A5 variants in familial neuropathic chronic itch. *Brain* 140, 555-567. 28073787 10.1093/brain/aww343

[DMM032946C61] Marvulli D., Volpin D. and Bressan G. M. (1996). Spatial and temporal changes of typeVI collagen expression during mouse development. *Dev. Dyn.* 206, 447-454. 10.1002/(SICI)1097-0177(199608)206:4<447::AID-AJA10>3.0.CO;2-U 8853993

[DMM032946C62] McComb R. D., Moul J. M. and Bigner D. D. (1987). Distribution of type VI collagen in human gliomas: comparison with fibronectin and glioma-mesenchymal matrix glycoprotein. *J. Neuropathol. Exp. Neurol.* 46, 623-633. 10.1097/00005072-198711000-00002 3655835

[DMM032946C63] Merlini L., Martoni E., Grumati P., Sabatelli P., Squarzoni S., Urciuolo A., Ferlini A., Gualandi F. and Bonaldo P. (2008). Autosomal recessive myosclerosis myopathy is a collagen VI disorder. *Neurology* 71, 1245-1253. 10.1212/01.wnl.0000327611.01687.5e 18852439

[DMM032946C64] Mohassel P., Rooney J., Zou Y. and Bönnemann C. (2015). Col6a2 null mice are a new mouse model of collagen-VI related dystrophies and relevant to the human disease. *Neuromuscul. Disord.* 25, S266 10.1016/j.nmd.2015.06.292

[DMM032946C65] Muona P., Jaakkola S., Zhang R. Z., Pan T. C., Pelliniemi L., Risteli L., Chu M. L., Uitto J. and Peltonen J. (1993). Hyperglycemic glucose concentrations up-regulate the expression of type VI collagen in vitro. Relevance to alterations of peripheral nerves in diabetes mellitus. *Am. J. Pathol.* 142, 1586-1597. 8494053 PMC1886917

[DMM032946C66] Neumann C., Yu A., Welge-Lüssen U., Lütjen-Drecoll E. and Birke M. (2008). The effect of TGF-β2 on elastin, type VI collagen, and components of the proteolytic degradation system in human optic nerve astrocytes. *Investig. Ophthalmol. Vis. Sci.* 49, 1464-1472. 10.1167/iovs.07-1053 18385064

[DMM032946C67] Oda Y., Kawahara E., Minamoto T., Ueda Y., Ikeda K., Nagai Y. and Nakanishi I. (1988). Immunohistochemical studies on the tissue localization of collagen types I, III, IV, V and VI in schwannomas: correlation with ultrastructural features of the extracellular matrix. *Virchows Arch. B Cell Pathol. Incl. Mol. Pathol.* 56, 153-163. 10.1007/BF02890013 2906188

[DMM032946C68] Palumbo C., Massa R., Panico M. B., Di Muzio A., Sinibaldi P., Bernardi G. and Modesti A. (2002). Peripheral nerve extracellular matrix remodeling in Charcot-Marie-Tooth type I disease. *Acta Neuropathol.* 104, 287-296. 12172915 10.1007/s00401-002-0558-0

[DMM032946C69] Pan T.-C., Zhang R.-Z., Markova D., Arita M., Zhang Y., Bogdanovich S., Khurana T. S., Bönnemann C. G., Birk D. E. and Chu M.-L. (2013). COL6A3 protein deficiency in mice leads to muscle and tendon defects similar to human collagen VI congenital muscular dystrophy. *J. Biol. Chem.* 288, 14320-14331. 10.1074/jbc.M112.433078 23564457 PMC3656288

[DMM032946C70] Pan T.-C., Zhang R.-Z., Arita M., Bogdanovich S., Adams S. M., Gara S. K., Wagener R., Khurana T. S., Birk D. E. and Chu M.-L. (2014). A mouse model for dominant collagen VI disorders: heterozygous deletion of Col6a3 Exon 16. *J. Biol. Chem.* 289, 10293-10307. 10.1074/jbc.M114.549311 24563484 PMC4036154

[DMM032946C71] Payano M., Kondo Y., Kashima K., Daa T., Yatsuka T., Kida H., Nakayama I. and Yokoyama S. (2004). Two cases of nondura-based clear cell meningioma of the cauda equina: case report. *APMIS* 112, 141-147. 10.1111/j.1600-0463.2004.apm1120209.x 15056231

[DMM032946C72] Peltonen J., Jaakkola S., Hsiao L. L., Timpl R., Chu M. L. and Uitto J. (1990). Type VI collagen. In situ hybridizations and immunohistochemistry reveal abundant mRNA and protein levels in human neurofibroma, schwannoma and normal peripheral nerve tissues. *Lab. Invest.* 62, 487-492. 2332972

[DMM032946C73] Peltonen J. T., Kalliomaki M. A. and Muona P. K. (1997). Extracellular matrix of peripheral nerves in diabetes. *J. Peripher. Nerv. Syst.* 2, 213-226. 10975727

[DMM032946C74] Perris R., Kuo H.-J., Glanville R. W. and Bronner-Fraser M. (1993). Collagen type VI in neural crest development: distribution in situ and interaction with cells in vitro. *Dev. Dyn.* 198, 135-149. 10.1002/aja.1001980207 8305706

[DMM032946C75] Quijano-Roy S., Renault F., Romero N., Guicheney P., Fardeau M. and Estournet B. (2004). EMG and nerve conduction studies in children with congenital muscular dystrophy. *Muscle Nerve* 29, 292-299. 10.1002/mus.10544 14755496

[DMM032946C76] Radev Z., Hermel J.-M., Elipot Y., Bretaud S., Arnould S., Duchateau P., Ruggiero F., Joly J.-S. and Sohm F. (2015). A TALEN-exon skipping design for a bethlem myopathy model in zebrafish. *PLoS ONE* 10, e0133986 10.1371/journal.pone.0133986 26221953 PMC4519248

[DMM032946C77] Ramanoudjame L., Rocancourt C., Lainé J., Klein A., Joassard L., Gartioux C., Fleury M., Lyphout L., Kabashi E., Ciura S. et al. (2015). Two novel COLVI long chains in zebrafish that are essential for muscle development. *Hum. Mol. Genet.* 24, 6624-6639. 10.1093/hmg/ddv368 26362255

[DMM032946C78] Rasi K., Hurskainen M., Kallio M., Stavén S., Sormunen R., Heape A. M., Avila R. L., Kirschner D., Muona A., Tolonen U. et al. (2010). Lack of collagen XV impairs peripheral nerve maturation and, when combined with laminin-411 deficiency, leads to basement membrane abnormalities and sensorimotor dysfunction. *J. Neurosci.* 30, 14490-14501. 10.1523/JNEUROSCI.2644-10.2010 20980607 PMC6634795

[DMM032946C79] Rogers S. L., Gegick P. J., Alexander S. M. and McGuire P. G. (1992). Transforming growth factor-β alters differentiation in cultures of avian neural crest-derived cells: effects on cell morphology, proliferation, fibronectin expression, and melanogenesis. *Dev. Biol.* 151, 192-203. 10.1016/0012-1606(92)90226-7 1577188

[DMM032946C80] Roggendorf W., Opitz H. and Schuppan D. (1988). Altered expression of collagen type VI in brain vessels of patients with chronic hypertension. *Acta Neuropathol.* 77, 55-60. 10.1007/BF00688243 3239376

[DMM032946C81] Sabatelli P., Gara S. K., Grumati P., Urciuolo A., Gualandi F., Curci R., Squarzoni S., Zamparelli A., Martoni E., Merlini L. et al. (2011). Expression of the collagen VI α5 and α6 chains in normal human skin and in skin of patients with collagen VI-related myopathies. *J. Invest. Dermatol.* 131, 99-107. 10.1038/jid.2010.284 20882040

[DMM032946C82] Sharma S., Ray S., Mukherjee S., Moiyadi A., Sridhar E. and Srivastava S. (2015). Multipronged quantitative proteomic analyses indicate modulation of various signal transduction pathways in human meningiomas. *Proteomics* 15, 394-407. 10.1002/pmic.201400328 25413884

[DMM032946C83] Sievers J., Pehlemann F. W., Gude S. and Berry M. (1994). Meningeal cells organize the superficial glia limitans of the cerebellum and produce components of both the interstitial matrix and the basement membrane. *J. Neurocytol.* 23, 135-149. 10.1007/BF01183867 8195812

[DMM032946C84] Solares Perez A., Gartioux C., Beuvin M., Thao M. V., Laine J., Medja F., Ferry A., Romero N. B., Bonne G. and Allamand V. (2012). G.P.18 Muscle pathology and dysfunction in a novel mouse model of COLVI-myopathy. *Neuromuscul. Disord.* 22, 827-828. 10.1016/j.nmd.2012.06.088

[DMM032946C85] Sollberg S., Muona P., Lebwohl M., Peltonen J. and Uitto J. (1991). Presence of type I and VI collagen mRNAs in endothelial cells in cutaneous neurofibromas. *Lab. Invest.* 65, 237-242. 1881124

[DMM032946C86] Soret R., Mennetrey M., Bergeron K. F., Dariel A., Neunlist M., Grunder F., Faure C., Silversides D. W. and Pilon N.; Ente-Hirsch Study Group. (2015). A collagen VI-dependent pathogenic mechanism for Hirschsprung's disease. *J. Clin. Invest.* 125, 4483-4496. 10.1172/JCI83178 26571399 PMC4665793

[DMM032946C87] Strekalova T., Sun M., Sibbe M., Evers M., Dityatev A., Gass P. and Schachner M. (2002). Fibronectin domains of extracellular matrix molecule tenascin-C modulate hippocampal learning and synaptic plasticity. *Mol. Cell. Neurosci.* 21, 173-187. 10.1006/mcne.2002.1172 12359159

[DMM032946C88] Telfer W. R., Busta A. S., Bonnemann C. G., Feldman E. L. and Dowling J. J. (2010). Zebrafish models of collagen VI-related myopathies. *Hum. Mol. Genet.* 19, 2433-2444. 10.1093/hmg/ddq126 20338942 PMC2876888

[DMM032946C89] Turtoi A., Blomme A., Bianchi E., Maris P., Vannozzi R., Naccarato A. G., Delvenne P., De Pauw E., Bevilacqua G. and Castronovo V. (2014). Accessibilome of human glioblastoma: collagen-VI-alpha-1 is a new target and a marker of poor outcome. *J. Proteome Res.* 13, 5660-5669. 10.1021/pr500657w 25325876

[DMM032946C90] Vitale P., Braghetta P., Volpin D., Bonaldo P. and Bressan G. M. (2001). Mechanisms of transcriptional activation of the col6a1 gene during Schwann cell differentiation. *Mech. Dev.* 102, 145-156. 10.1016/S0925-4773(01)00303-3 11287188

[DMM032946C91] Von Kaisenberg C. S., Brand-Saberi B., Vallian S., Farzaneh F. and Nicolaides K. H. (1998). Collagen type VI gene expression in the skin of trisomy 21 fetuses. *Obstet. Gynecol.* 91, 319-323. 10.1016/S0029-7844(97)00697-2 9491853

[DMM032946C92] Wallquist W., Plantman S., Thams S., Thyboll J., Kortesmaa J., Lännergren J., Domogatskaya A., Ogren S. O., Risling M., Hammarberg H. et al. (2005). Impeded interaction between Schwann cells and axons in the absence of laminin alpha4. *J. Neurosci.* 25, 3692-3700. 10.1523/JNEUROSCI.5225-04.2005 15814800 PMC6725372

[DMM032946C93] Weinreb R. N., Aung T. and Medeiros F. A. (2014). The pathophysiology and treatment of glaucoma: a review. *JAMA* 311, 1901-1911. 10.1001/jama.2014.3192 24825645 PMC4523637

[DMM032946C94] Yagihashi S., Yamagishi S.-I. and Wada R. (2007). Pathology and pathogenetic mechanisms of diabetic neuropathy: correlation with clinical signs and symptoms. *Diabetes Res. Clin. Pract.* 77 Suppl. 1, S184-S189. 10.1016/j.diabres.2007.01.054 17462777

[DMM032946C95] Yang D., Bierman J., Tarumi Y. S., Zhong Y.-P., Rangwala R., Proctor T. M., Miyagoe-Suzuki Y., Takeda S., Miner J. H., Sherman L. S. et al. (2005). Coordinate control of axon defasciculation and myelination by laminin-2 and -8. *J. Cell Biol.* 168, 655-666. 10.1083/jcb.200411158 15699217 PMC2171752

[DMM032946C96] You W.-K., Bonaldo P. and Stallcup W. B. (2012). Collagen VI ablation retards brain tumor progression due to deficits in assembly of the vascular basal lamina. *Am. J. Pathol.* 180, 1145-1158. 10.1016/j.ajpath.2011.11.006 22200614 PMC3349878

[DMM032946C97] Yu L., Xie B., Yin X., Liang M., Evans A. C., Wang J. and Dai C. (2013). Reduced cortical thickness in primary open-angle glaucoma and its relationship to the retinal nerve fiber layer thickness. *PLoS ONE* 8, e73208 10.1371/journal.pone.0073208 24019910 PMC3760921

[DMM032946C98] Zech M., Lam D. D., Francescatto L., Schormair B., Salminen A. V., Jochim A., Wieland T., Lichtner P., Peters A., Gieger C. et al. (2015). Recessive mutations in the α3(VI) collagen gene COL6A3 cause early-onset isolated dystonia. *Am. J. Hum. Genet.* 96, 883-893. 10.1016/j.ajhg.2015.04.010 26004199 PMC4457951

[DMM032946C99] Zhang W. W. and Olsson Y. (1997). The angiopathy of subcortical arteriosclerotic encephalopathy (Binswanger's disease): immunohistochemical studies using markers for components of extracelluar matrix, smooth muscle actin and endothelial cells. *Acta Neuropathol.* 93, 219-224. 10.1007/s004010050607 9083552

[DMM032946C100] Zhang W. W., Lempessi H. and Olsson Y. (1998). Amyloid angiopathy of the human brain: immunohistochemical studies using markers for components of extracellular matrix, smooth muscle actin and endothelial cells. *Acta Neuropathol.* 96, 558-563. 10.1007/s004010050935 9845284

